# Non-Local Means Inpainting of MS Lesions in Longitudinal Image Processing

**DOI:** 10.3389/fnins.2015.00456

**Published:** 2015-12-15

**Authors:** Nicolas Guizard, Kunio Nakamura, Pierrick Coupé, Vladimir S. Fonov, Douglas L. Arnold, D. Louis Collins

**Affiliations:** ^1^McConnell Brain Imaging Center, Montreal Neurological Institute, McGill UniversityMontreal, QC Canada; ^2^Department of Biomedical Engineering, Lerner Research Institute, Cleveland ClinicCleveland, OH, USA; ^3^Pictura Research Group, Unité Mixte de Recherche Centre National de la Recherche Scientifique, Laboratoire Bordelais de Recherche en InformatiqueTalence, France

**Keywords:** MS, lesions, segmentation, inpainting, non-local, patch-based, MRI

## Abstract

In medical imaging, multiple sclerosis (MS) lesions can lead to confounding effects in automatic morphometric processing tools such as registration, segmentation and cortical extraction, and subsequently alter individual longitudinal measurements. Multiple magnetic resonance imaging (MRI) inpainting techniques have been proposed to decrease the impact of MS lesions in medical image processing, however, most of these methods make the assumption that lesions only affect white matter. Here, we propose a method to fill lesion regions using the patch-based non-local mean (NLM) strategy. The method consists of a hierarchical concentric filling strategy after identification of the lesion region. The lesion is filled iteratively, based on the surrounding tissue intensity, using an onion peel strategy. This concentric technique presents the advantage of preserving the local information and therefore the continuity of the anatomy and does not require identification of any a priori normal brain tissues. The method is first evaluated on 20 healthy subjects with simulated artificial MS lesions where we assessed our technique by measuring the peak signal-to-noise ratio (PSNR) of the images with inpainted lesion and the original healthy images. Second, in order to assess the impact of lesion filling on longitudinal image analyses, we performed a power analysis with sample size estimation to evaluate brain atrophy and ventricular growth in patients with MS. The method was compared to two different publicly available methods (FSL lesion fill and Lesion LEAP) and a more classic method, which fills the region with intensities similar to that of the surrounding healthy white matter tissue or mask the lesions. The proposed method was shown to exceed the other methods in reproducing the fidelity of healthy subject images where the lesions were inpainted. The method also improved the power to detect brain atrophy or ventricular growth by decreasing the sample size by 25% in the presence of MS lesions.

## Introduction

Multiple sclerosis (MS) is a chronic autoimmune disease that affects the central nervous system (CNS) and presents different clinical variants but it usually starts with a relapsing remitting phase (RRMS). The underlying neuronal pathology of a relapse consists of attacks of the myelin and creates focal inflammation leading to lesions in both white matter (WM) and gray matter (GM) and can ultimately lead to demyelination, gliosis and axonal loss. Quantification of MS lesions, also known as plaques, is often used in clinical studies as a marker for disease burden because they are visible on conventional magnetic resonance imaging (MRI) (Fazekas et al., 1999). In addition, MRI enables the exploration of the morphological differences. In MS, structural segmentation (i.e., tissue classification, Zijdenbos et al., 1998) and voxel-wise analysis [i.e., voxel-based morphometry (VBM; Prinster et al., 2006; Lansley et al., 2013)] or deformation based-morphometry (DBM; Tao et al., 2009) have been used to measure these differences. These tools have been used to assess longitudinal changes of anatomical structures (Nakamura et al., 2014) or normal appearing brain tissue (NABT; Sanfilipo et al., 2006). However, MS lesions can swell, shrink and disappear over weeks or months depending on the pathological activity and evolution of the disease (Rovira et al., 2013). These longitudinal changes affect their appearance on MRI and thus can potentially affect image processing tools such as registration (Brett, 2001; Meier and Fisher, 2005) and tissue classification (Nakamura and Fisher, 2009; Chard et al., 2010), and may lead to longitudinal inconsistencies.

In order to remove the variability due to MS lesions, various approaches have been proposed. Depending on the application and the final objective, after identification of the region of interest (ROI), it is possible either to remove (“Mask-out”) or to replace these voxels with potential NABT intensity values. Masking-out MS lesion has shown some limitations in the context of longitudinal brain atrophy measurements (Battaglini et al., 2012). Lesion filling or inpainting strategies consist in replacing or synthesizing voxel values within the region of the MS lesion by representative NABT values. A variety of approaches have been proposed in the literature. Sdika and Pelletier (2009) described three different inpainting strategies: *basic, local white matter (LWM), and global white matter inpainting*. *Basic inpainting* was inspired from Telea (2004) and consists in propagating the local average of the outer region toward the inner region of the lesion mask equivalent to an *onion peel strategy*. *Local white matter inpainting* uses a prior tissue classification of the NABT to fill the lesion with the local normal appearing WM (NAWM) intensity average. *Global white matter inpainting* fills the lesion region with the global intensity average of the NAWM obtained from the tissue classification. Chard et al. (2010) proposed LEAP (LEsion Automated Preprocessing) which also uses NABT classification but extracts the NAWM histogram properties to obtain its intensity peak and noise properties to fill the lesion region. Later, Battaglini et al. (2012) proposed an approach implemented in FSL^1^ which fills the lesion with random intensity values from the surrounding NABT distribution of WM and partial WM volumes. These methods focused on reducing the impact of white matter lesions and have been shown to improve results for cortical GM atrophy measurement (Ceccarelli et al., 2012; Magon et al., 2014; Popescu et al., 2014) as well as for white matter atrophy estimation (Chard et al., 2010). However, methods such as *basic inpainting* use the surrounding voxels to fill and propagate intensities and thus can potentially fill the lesion regions with undesired intensities. The main limitation of these methods is their assumption that only WM should contain lesions. Furthermore, these methods rely on tissue classification which can be challenging in presence of MS (Derakhshan et al., 2010) due to the underlying neuropathology affecting the NAWM intensity (Vrenken et al., 2006).

In the computer vision community, the field of image inpainting has the goal of producing a plausible image after the removal of a region defined by an operator. Inpainting is often used to restore image deterioration (e.g., scratches, dust speckles…), remove or add elements (e.g., text elements, publicities, persons…) from the remaining information of the image. The main inpainting methods in the literature may be categorized as being sparsity-based, variational, and patch-based. Bertalmío et al. (2014) provides an interesting review of the inpainting literature. Here we describe a patch-based approach inspired from methods that were initially proposed for texture synthesis. During the last few decades, several paradigms have been used in computer vision. First, the method described in Efros and Leung (1999) has proven to be effective, using an “onion-peel” strategy to fill the region from its outer surface to its inner core. Their method compares the available patches (small regions of the image) and fills the considered “empty” central voxel of a patch (a small nxn area, where typically *n* = 5.15) with the central voxel intensity value of the most similar patch before moving to the next voxel to be filled. Later, Criminisi et al. (2004) proposed an *exemplar-based* approach which fills the whole patch instead of the central voxel for faster processing, while prioritizing the filling of edges first. Despite impressive visual results, several limitations remain for these inpainting algorithms. The main limitation is that by using only the best match sample chosen could be corrupted or not a perfect match. More recently, the Non-Local Mean (NLM) method, used to compare patch similarities initially proposed for image denoising (Buades et al., 2005), takes advantage of the image redundancy by using a large number of patches instead of the closest one and has been applied to 2D image inpainting (Wong and Orchard, 2008).

Although using patch-based inpainting strategies has shown promising results in computer vision in natural and artificial scenes, it has yet not been fully exploited in medical imaging. This approach presents the enormous advantage of not requiring any tissue segmentation *a priori*, and allowing rough larger lesion delineations. Another advantage of not requiring tissue classification is that the method does not depend on specific image contrasts. Indeed, our inpainting approach can be applied to any types of MRI acquisition protocols. Inspired by the computer vision inpainting techniques, we used an exemplar-based NLM inpainting strategy in the context of MS lesion filling in MRI (Guizard et al., 2013). The proposed method consists of a concentric filling strategy. After identification of the lesion region, the lesion is filled using an onion peel strategy where concentric layers' voxels of the lesion are successively replaced by the weighted average of the surrounding *normal* patches (detailed below). Inspired by our initial NLM lesion inpainting technique (Guizard et al., 2013), Prados et al. (2014) applied a similar approach with a different initialization strategy (they used the original voxel values) and smoothed the NLM result. Here, we propose to improve the initialization and the convergence using a hierarchical framework, which synthesizes the image intensity variability in the lesion mask.

In this article, we provide a thorough validation using simulated lesions on healthy subjects where we assessed the similarity of the inpainted lesion images and the original images using peak signal noise ratio (PSNR). We also performed power analysis on longitudinal MS patient data to detect changes over time. We compare our proposed method to three different publicly available MS inpainting methods: LWMI (Sdika and Pelletier, 2009), LEAP (Chard et al., 2010), and FSL lesion filling (Battaglini et al., 2009).

## Methods

In the following section we first describe the NLM inpainting, the Filling Strategy and the proposed hierarchical approach. Here, given an image *I* and the lesion ROI (*L*), we define the inpainted image Î at the voxel location *i* as to obtain the final image I^*^, such as:

(1) $$I^{*}(i)=\{\begin{array}{c} \hat{I}(i)\forall i\in L(i)\\ I(i)\forall i\notin L(i). \end{array}$$

### NLM inpainting

The propose NLM inpainting approach takes advantage of image redundancy to locally average similar realizations of the image. Indeed, the idea of the NLM was initially proposed for image denoising (Buades et al., 2005) to reduce the noise of the image by averaging the voxels of patches that would have the same intensity in the noise-free image. Similarly to denoising, our inpainting strategy exploits the redundancy of the image to fill the lesion.

**Figure 1:**
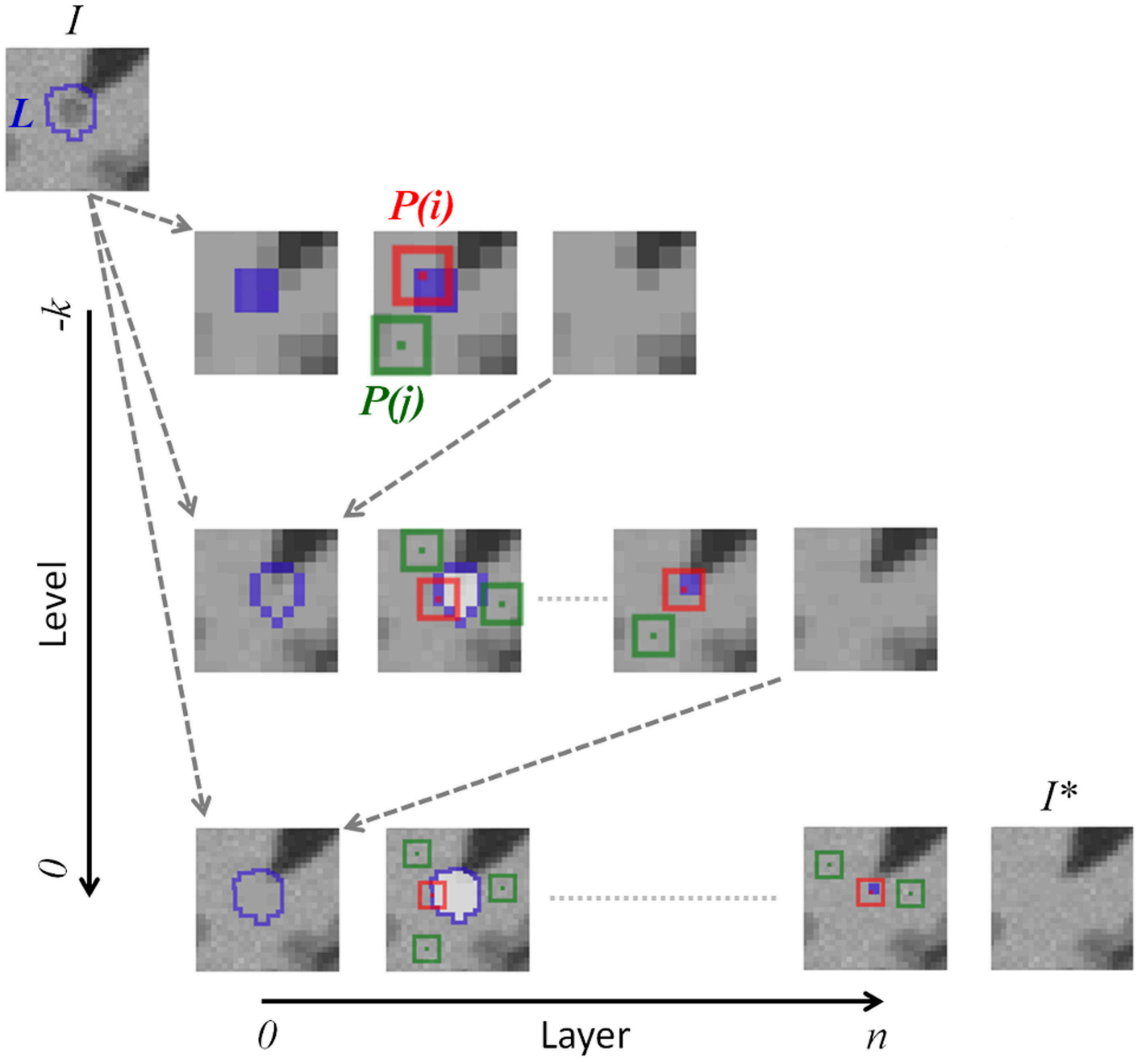
**NLM lesion inpainting strategy**. The inpainting process starts with the lesion mask (*L*) of the original image (*I*) in the downsampled space *k* to obtain the inpainted image of this level. Then, the inpainted region is upsampled into the image of the next hierarchical level. The inpainting itself consists in finding the most similar patches [*P(j)*, in green] in the “non-lesion” region with the considered patch *P(i)*. All voxels in white are not considered during the patch distance estimation. The concentric filling is described by the boundary of the current mask (L^*^ in yellow) shrinking by one voxel at the next “Layer”. The original lesion mask *L* is reinitiated at the beginning of each hierarchical “Level.”

The patch distance estimator (*dist*) used for denoising is here adapted for inpainting by comparing the patch *P(I(i))* centered on *i* (in red in Figure 1) with the patch *P(I(j))* centered on *j* (in green in Figure 1) within a certain search area (Ω):

(2) dist(P(I(i)),P(I(j)))= ∑x∈P(I(i))y^∈P(I(j))|i∈L*^jϵΩ (I(x)−I(y))2

where the voxel *i* belongs to the considered lesion mask layer L^*^ (in yellow in Figure 1).

This distance is then used to computed the weighted function, *w(i,j)*, designed to attribute a smaller weight to greater distance measures of the corresponding patches *P(I(i))* and *P(I(j))*, such as:

(3) $$w\left(i,j\right)=e^{-\frac{dist\left(P\left(\hat{I}\left(i\right)\right),P\left(\hat{I}\left(j\right)\right)\right)}{h^{2}}}$$

where *h*^2^ is a chosen smoothing parameter, discussed in the following section.

Once the corresponding distance with *i* of every voxel *j* belonging to Ω is estimated, the ROI, L^*^(x), is filled with the weighted average:

(4) $$\hat{I}{\left(i\right)}_{i\in L^{\text{*}}}=\frac{\sum_{j\epsilon \Omega }w\left(i,j\right)I\left(j\right)}{\sum_{j\epsilon \Omega }w\left(i,j\right)}$$

### Filling strategy

The filling strategy is important in image inpainting in order to preserve continuity of edges and visual consistency present in the true image. The proposed NLM inpainting strategy consists in a hierarchical inpainting of concentric layers, iterated for different smoothing parameters.

#### Concentric filling

The concentric technique presents the advantage of propagating the local information and therefore the continuity of the anatomy (Efros and Leung, 1999).

Cubic patches of voxels from the outermost layer to be filled are compared to patches from the surrounding voxels not in the lesion mask *L*. After filling a layer of *L*, this process is repeated on the next interior layer of the new lesion mask, L^*^, until reaching its core. Only voxels outside of L^*^, thus including the already inpainted voxels, are used during the patch distance estimation.

#### Hierarchical inpainting

In order to optimize the performance of the NLM inpainting and to reduce the ambiguity in the case of large lesions (Liu and Caselles, 2013), we embed the filling strategy within a hierarchical multi-resolution framework.

Starting from the downsampled resolution and the outside layer of the lesion mask, the process fills the next interior layer until reaching the center of the lesion mask before moving to the next hierarchical level where this process is repeated. The original image and its lesion mask are interpolated at different resolution scales (*k*) using, respectively, tri-linear and nearest neighbor interpolations. Starting from the lowest resolution level, the inpainting results of the innermost concentric layer are then used to initialize the following level. The inpainted regions of lower *k* levels are interpolated using tri-linear interpolation to the *k-1 level* to replace the voxels filled at the previous iteration.

#### Smoothing parameter (*h*^2^)

Within the NLM approaches, *h*^2^ is critical to attribute weight to the most similar patches. For our inpainting problem, decreasing *h*^2^ attributes less weight to less similar patches while a bigger *h*^2^-value tends to provide smoother inpainting results. Therefore, for each inpainted voxel at each hierarchical level and each concentric layer, we iterate the NLM inpainting with the following successive *h*^2^-values [0.9, 0.7, 0.5, 0.3, 0.1]. Starting the inpainting of the considered voxel with a big *h*^2^, we initiate the voxel filling with a smooth value with respect to the neighborhood (Ω). Then, successively decreasing *h*^2^ to 0.1 is equivalent to searching for the most similar patch (i.e., the minimum intensity distance) in Ω, thus synthesizing the finer image textural details.

The concentric and hierarchical inpainting processes are graphically illustrated, respectively, by the “Layer” and the “Level” axes in Figure 1. In the following experiments, we used three (*k* = 3) isotropic resolution levels (4, 2, and 1 mm) with similar patch sizes (9 × 9 × 9 voxels).

## Experiments

In the following section we describe (1) the data used in our experiments, (2) the simulated MS lesion data such that the original MRI intensity information can be used as a ground truth, (3) the longitudinal power analysis to detect brain atrophy, and (4) the different methods evaluated.

### Data

The Montreal Neurological Institute research ethics committee gave approval for this study and all subjects gave informed consent. To evaluate the proposed algorithm, two neuroimaging datasets were used anonymously:

- From a multi-site clinical study with 67 relapsing-remitting MS patients (RRMS, mean age 37.5 y, SD 10.0 y). Each patient underwent an MRI at two time points, baseline (*m00*) and 12 month (*m12*), that included sagittal T1W (*TE* = 9−11 ms, *TR* = 30−40 ms, flip angle = 30°, in-plane resolution = 0.977 × 0.977 mm^2^, slice thickness = 1.5 mm), T2W (*TE* = 65−104 ms, *TR* = 3666−8585 ms, flip angle = 90°, in-plane resolution = 0.977 × 0.977 mm^2^, slice thickness = 3 mm), and PD (*TE* = 10−18 ms, *TR* = 2200−3800 ms, flip angle = 90°, in-plane resolution = 0.977 × 0.977 mm^2^, slice thickness = 3 mm) images. The MRI data were acquired on 1.5T scanners from different manufacturers: GE (*n* = 20), Philips (*n* = 18), and Siemens (*n* = 29).
- From this RRMS database, we randomly selected T1W images of 20 MS patients to simulate realistic MS lesions on BrainWeb simulation MRIs (http://brainweb.bic.mni.mcgill.ca/brainweb/; Collins et al., 1998) from 20 healthy subjects (Aubert-Broche et al., 2006).

Although the application of our inpainting method is not limited to a specific imaging modality, T1W images were chosen since they are acquired as part of many standard imaging protocols and are widely used to assess longitudinal volume changes in MRI. In addition, this modality was used by the other inpainting methods we wish to compare to in this analysis.

### Artificial MS lesions validation

The different inpainting methods are evaluated using artificial numerical MS lesions that are simulated on healthy subject MRIs. Simulations are done using the strategy of Brett (2001), whereby MRI data from MS patients are used to simulate WM lesions on healthy subject MRIs. Here, the goal was to create T1W MS lesions for which we know the underlying ground truth (from the healthy subject data) such that we can compare inpainting results across different methods.

**Figure 2:**
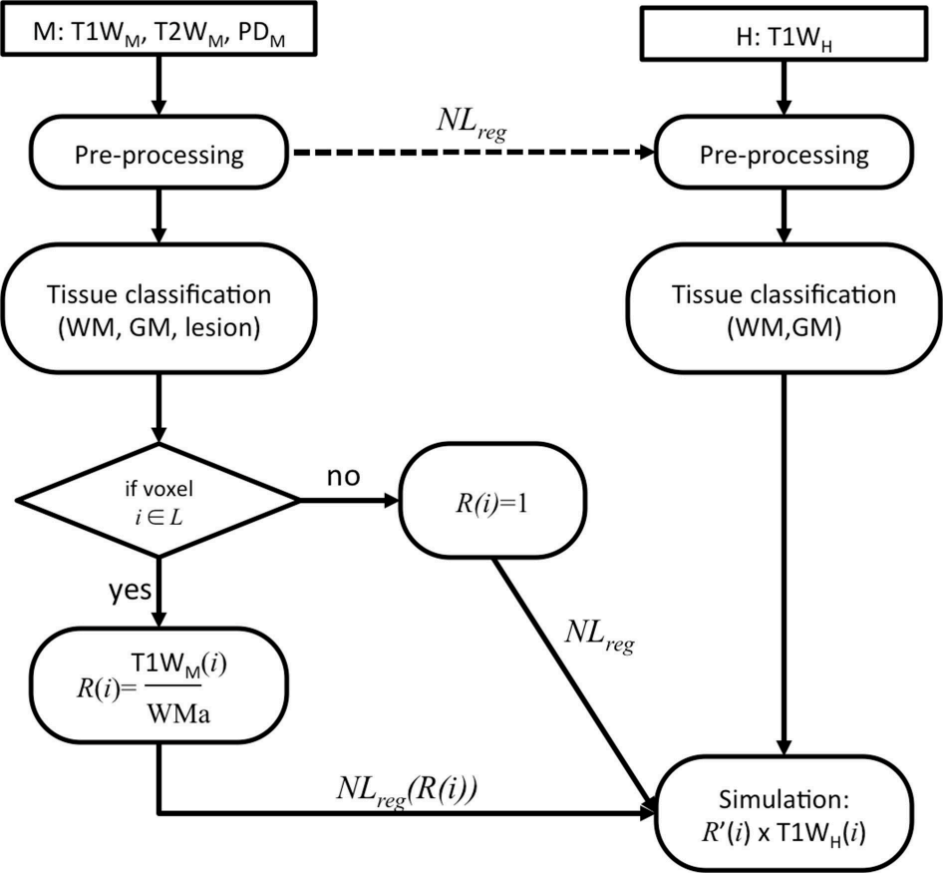
**Flowchart of the lesion simulation**.

The simulation, illustrated in Figure 2, was performed on the healthy brain image (*H*) using real lesions from the MS patient image (*M*), and can be summarized as follows:

1. Pre-processing: (i) intensity non-uniformity correction (Sled et al., 1998), (ii) intensity normalization using linear histogram matching, and (iii) linear registration (Collins et al., 1994) to the stereotaxic ICBM152 template.
2. Tissue classification of *H* and *M*: after an automatic segmentation of the WM, GM, cerebrospinal fluid (CSF) and T2W MS lesions (only on the patients) by a multi-spectral Bayesian classifier (Francis, 2004) using the T1W, T2W, and PD images. From prior probability model of the segmentation estimated from a training dataset, the *M* is segmented using Bayes' theorem, where the distribution of each tissue classes is used to estimate the parameters of their Gaussian distribution. The automatic T2W lesion outlines of *M* were superimposed on T1W, T2W, and PD for manual reviews. Experts who underwent extensive training on similar MS patient MRI data carefully reviewed the MS lesion mask, *L*.
3. For each H:M pair: Compute the voxel-wise intensity ratio (*R*) of the healthy WM (obtained from stage 2) intensity average (WMa) and the T1W voxel intensity of lesion tissue [T1W_M_(*i*)] from the corresponding manually-corrected mask (*L*) of *M* for a voxel *i*:
  (5) $$R_{M}\left(i\right)=\{\begin{array}{c} \frac{{\text{T1W}}_{\text{M}}(i)}{\text{WMa}}\\ \text{1} \end{array}\;\begin{array}{c} \forall \text{i}\in L\\ \forall \text{i}\notin L \end{array}$$
4. Estimate the non-linear transformation (*NL*_*reg*_) between *M* and *H* (Avants et al., 2008).
5. Using the transformation (*NL*_*reg*_), interpolate spatially *R* and *L* into the *H* space and obtain *R'* and *L'*.
6. From the interpolated *R'* and *L'*, create a new image (*H'*) where the final image intensity voxels equal *R'* × *H* where the lesion (*L'*) is defined and *H* everywhere else.

The six steps are repeated for the 20 H:M pairs. The resulting simulated dataset allowed us to assess the impact of the patch search radius for the proposed NLM inpainting before comparing it to state of the art inpainting approaches.

As a means to evaluate the inpainting algorithms, we can assess the fidelity of the restored image I^*^ by comparing it to the original image *I*. In the computer vision literature (VQEG, 2003; Wong and Orchard, 2008; Fadili et al., 2009), this is often done using the peak signal to noise ratio (PSNR) by measuring the ratio of the maximum possible power of the signal and the mean squared error (MSE) between the restored and the original image:

(6) $$PSNR=20.log_{10}\left(\frac{MAX_{1}}{\sqrt{MSE}}\right)$$

where *MAX*_1_ is themaximum possible pixel value (255 for 8 bits voxel storage) while MSE is estimated within the lesion mask, *L*, between the original image *I* (before adding the lesion) and the inpainted version, I^*^:

(7) $$MSE=\frac{1}{n}\sum_{x=1\in L}^{n}{\left\|I^{*}\left(x\right)-I\left(x\right)\right\|}^{2}$$

where *n* is the number of voxels. Thus, in this MS lesion simulation framework, we expect a smaller MSE, and thus a higher PSNR when the reconstruction is more similar to the original image.

We first evaluated the performance of our NLM inpainting approach for different search area radii, which is an important factor to find similar patches. The size of the search radius also influences the computational burden.

We then assessed the PSNR results of the different inpainting methods while simulating potential manual lesion segmentation variability and lesion mask misalignment. This is done by varying the original lesion mask boundary (β_0_) with morphological operations on the ground truth lesion mask, through dilation by 1 or 2 voxel layers (β_1_ and β_2_) around the whole lesion mask volume. This will enable characterization of the methods with respect to smaller or larger lesions.

### Longitudinal MS data validation

In the second set of experiments, our proposed inpainting technique is evaluated and compared to three different publicly available inpainting algorithms and a masking technique using real longitudinal MS patient data in order to determine the impact of the method on the power to detect longitudinal volume changes.

The longitudinal MRIs were pre-processed using steps (1) and (2) of Section Longitudinal MS Data Validation in order to obtain the lesion segmentations for each subject's time-point. All inpainting techniques used to the same set of lesion labels for the comparison.

In order to compare the performance of the inpainting methods, the popular longitudinal atrophy measurement tool SIENA (Smith et al., 2002) was used to measure the percent brain volume change (PBVC) as well as the percent ventricular volume change (PVVC) in the MS dataset. SIENA starts with brain and skull segmentation (Smith, 2002) to perform skull-based registration and analysis in the half-way space of the subject. Then, the brain and non-brain boundary is estimated from tissue classification (Zhang et al., 2001) before computing the perpendicular displacement between the brain boundaries of the two time-points. Finally, the surface displacement is averaged to obtain a global estimate of PBVC, and the PVVC if ventricle masks are used instead of brain masks.

Statistical comparison of the inpainting approaches was conducted using power analysis where we estimated the sample size (*per arm*), *n*, required to detect pre-specified treatment effect without accounting for normal aging atrophy (Anderson et al., 2007), such as:

(8) $$n=\frac{\text{2}{\left[\left(a\;+\;b\right)\right]}^{2}\sigma^{2}}{{\left(\mu_{1}-\mu_{2}\right)}^{2}}$$

where μ_1_ and μ_2_ are the mean rate of volume change in the placebo and treated groups, respectively, and σ^2^ the corresponding variance of the rate of change. Here, we only had a control MS group, we thus estimated sample sizes for 10, 30, and 50% treatment effects, so that *u*_2_ = *u*_1_ * (1 − 0.10), *u*_2_ = *u*_1_ * (1 − 0.30), and *u*_2_ = *u*_1_ * (1 − 0.50), respectively. The analysis was conducted with 80% power (*a* = 0.842) and a significance level of 0.05 (*b* = 1.96). The 95% confidence intervals were estimated by bootstrapping 10,000 times. The treatment effect are derived from previous clinical trial studies, where treatment effects on RRMS brain atrophy was around 50% (Rudick et al., 1999).

### Methods compared

We compared our method to 4 other methods that deal with MS lesions: 3 inpainting methods and 1 masking method:

- **LWM** (Sdika and Pelletier, 2009) estimates the tissue classes of the NABT to fill the lesion with the intensity average of the surrounding NAWM. Because this method is not publically available, we implemented our own version.
- **LEAP** (Chard et al., 2010) also uses tissue classification of the NABT but applies the intensity properties of the NAWM histogram to the region being filled. LEAP is available at: http://www.nmrgroup.ion.ucl.ac.uk/analysis/lesionfill.html.
- **FSL** lesion filling (Battaglini et al., 2009) fills the lesion from random intensity values estimated in the surrounding NABT after estimating the tissue WM and partial WM volumes. The **FSL** lesion filling method is available at: http://fsl.fmrib.ox.ac.uk/fsl/fslwiki/lesion_filling.
- **Masked-out**: We also evaluated the impact of removing the MS-lesion for the longitudinal analysis by masking the lesion out (or so called “**Masked-out**” approach, Battaglini et al., 2012).

## Results

### Artificial MS lesions

#### NLM inpainting search radius

**Figure 3:**
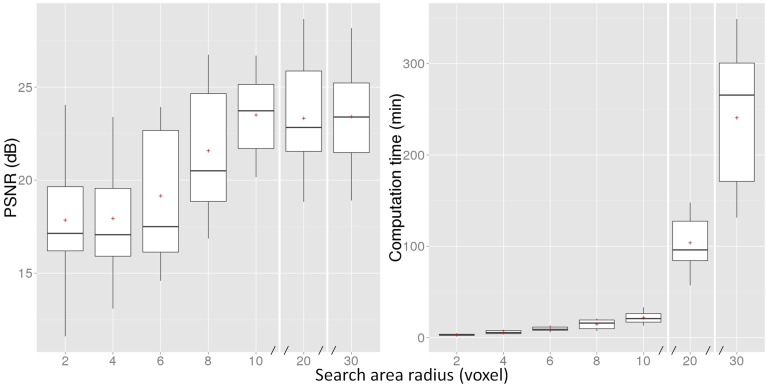
**PSNR measure and computation time of the proposed NLM method for different search area radii**. The boxes represent the lower and upper quartile with the median as the central black line and the mean with a red cross. The whiskers extend to the most extreme data point. Note that the x-axis is discontinuous after 10 voxels.

The NLM inpainting algorithm does not require *a-priori* knowledge of the NABT, GM, or WM and searches for the most similar patches throughout the whole brain. However, as shown in Figure 3, the PSNR plateaus around a radius of 10 voxels (note the discontinuous × axes), precluding the need of doing a brain-wide search. As such, a search area radius of 10 voxels was used in the remaining experiments since it provides a good compromise between reconstruction fidelity and computational burden.

#### Inpainting of simulated MS lesions

Here, we compare the NLM inpainting approach with LWM, FSL, and LEAP, while incorporating segmentation variability by simulating different lesion boundaries from the original lesion segmentation (β_0_). We do not compare to the Masking-out technique, as it does not attempt to model the original data.

**Figure 4:**
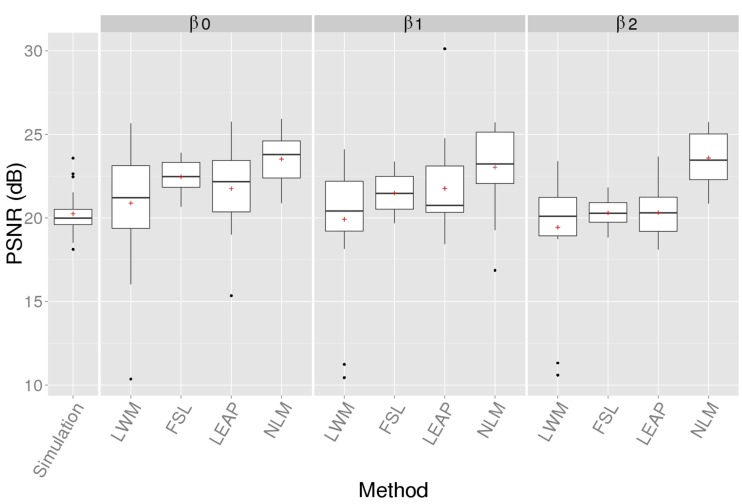
**PSNR of the simulated (no-inpainting) and inpainted images with the 4 techniques when compared to the original images for different lesion mask boundaries (β)**. Statistical analysis at β0 is reported in Table 1.

Figure 4 presents the PSNR results of the inpainting strategies for 3 different levels of lesion mask boundaries. We can notice that NLM outperforms the other methods regardless of the lesion mask size (β_0_, β_1_, and β_2_).

A One-way between subjects ANOVA was conducted to compare the effect of inpainting on the PSNR reconstruction measure [*F*_(4, 135)_ = 6.40, *p* < 0.01]. The Bonferroni-adjusted *t*-test analysis revealed that NLM is significantly better than LWM and LEAP (*p* < 0.01) with β_0_ and these results are summarized in Table 1.

**Table 1:** **Mean average and standard deviation (***SD***) of the PSNR for the simulated images without inpainting (no-inpainting) and the different inpainting methods with the original lesion mask (β_0_) and Bonferroni-adjusted multi-comparison ***t***-test of the PSNR results**.

	**Simulation (no-inpainting)**	**LWM**	**FSL**	**LEAP**	**NLM**
Mean PSNR	20.25	20.89	22.46	21.75	**23.52**
*SD*	1.38	3.47	1.01	2.33	**1.42**
*t*-test with NLM [(*t*-value, df), *p*-value]	[(8.68, 27), < 0.01]	[(5.32, 27), < 0.01]	[(2.56, 27), 0.03]	[(5.43, 27), < 0.01]	

*The bold score represent the highest mean PSNR measure*.

NLM's PSNR is stable when β increases since this approach is not specific to WM intensity distribution which can be altered when the mask used to compute the PSNR becomes bigger than the actual simulated lesion.

**Figure 5:**
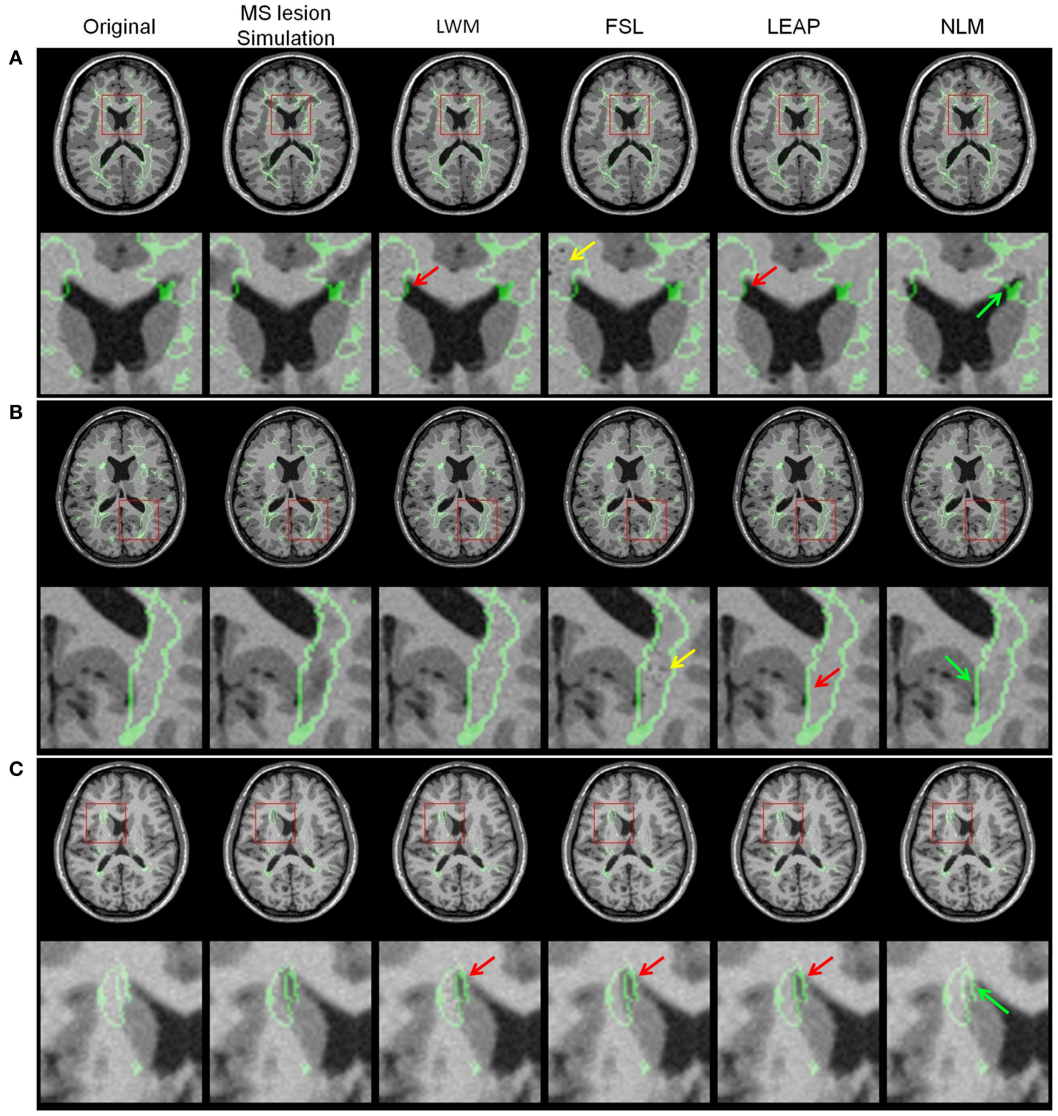
**Lesion simulation examples with the original image, MS lesion simulation and the different lesion filling results LWM, FSL, LEAP, and NLM for three different lesion sizes [(A) = large, (B) = medium and (C) = small]**. The red arrows point to anatomically improbable lesion filling results, the yellow arrows point to intensity problems while the green arrows point to plausible and realistic anatomy. The original lesion boundary and the red square on the axial image depicts the zoom-in image region shown below it. Note that these images were generated at β0.

Figure 5 illustrates examples of the inpainting results for the different techniques based on original images and the simulation of three different lesion types. The 3 cases were chosen to visualize typical large **(A)**, medium **(B)**, and small **(C)** peri-ventricular MS lesions. Visual inspection of the lesion filling with NLM shows qualitatively more plausible contrast, intensity gradients, texture and anatomy compared to other methods. For example, in case A, the NLM inpainting recovers the curved contour of the lateral ventricles despite the fact that the lesion mask reaches the CSF boundary. This is not the case for the LWM and LEAP methods; both show some “bleeding” into the ventricles (red arrows in second row of Figure 5). In addition, on cases Figures 5B,C, the WM/GM boundary gradient is more gradual with NLM and more faithfully reproduces the original contrast. Furthermore, the overall texture of the NLM reproduces the surrounding noise level, while LEAP tends to over-smooth and FSL seems to introduce noise (black dots highlighted with yellow arrows in cases Figures 5A,B).

**Figure 6:**
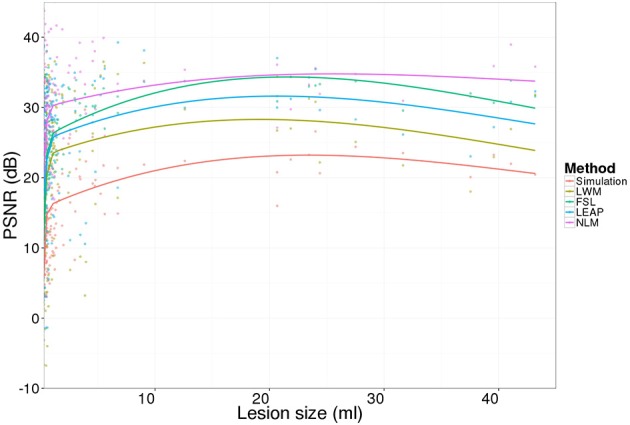
**Lesion-wise PSNR results for the 20 simulated images (no-inpainting) and the inpainting approaches (LWM, FSL, LEAP, and NLM)**.

Finally, NLM presents consistently better PSNR results regardless of the lesion size as we can appreciate on Figure 6. The simulated lesion PSNR increases with lesion size due to the simulation limitation which simulates T1W lesions from the original lesion mask obtained for T2W lesion resulting in generally “bigger” lesion masks. Therefore, this over segmentation results in simulated tissue intensities that are similar to the healthy tissue (Figure 5).

### Longitudinal MS data

#### Lesion inpainting on an individual MS longitudinal dataset

**Figure 7:**
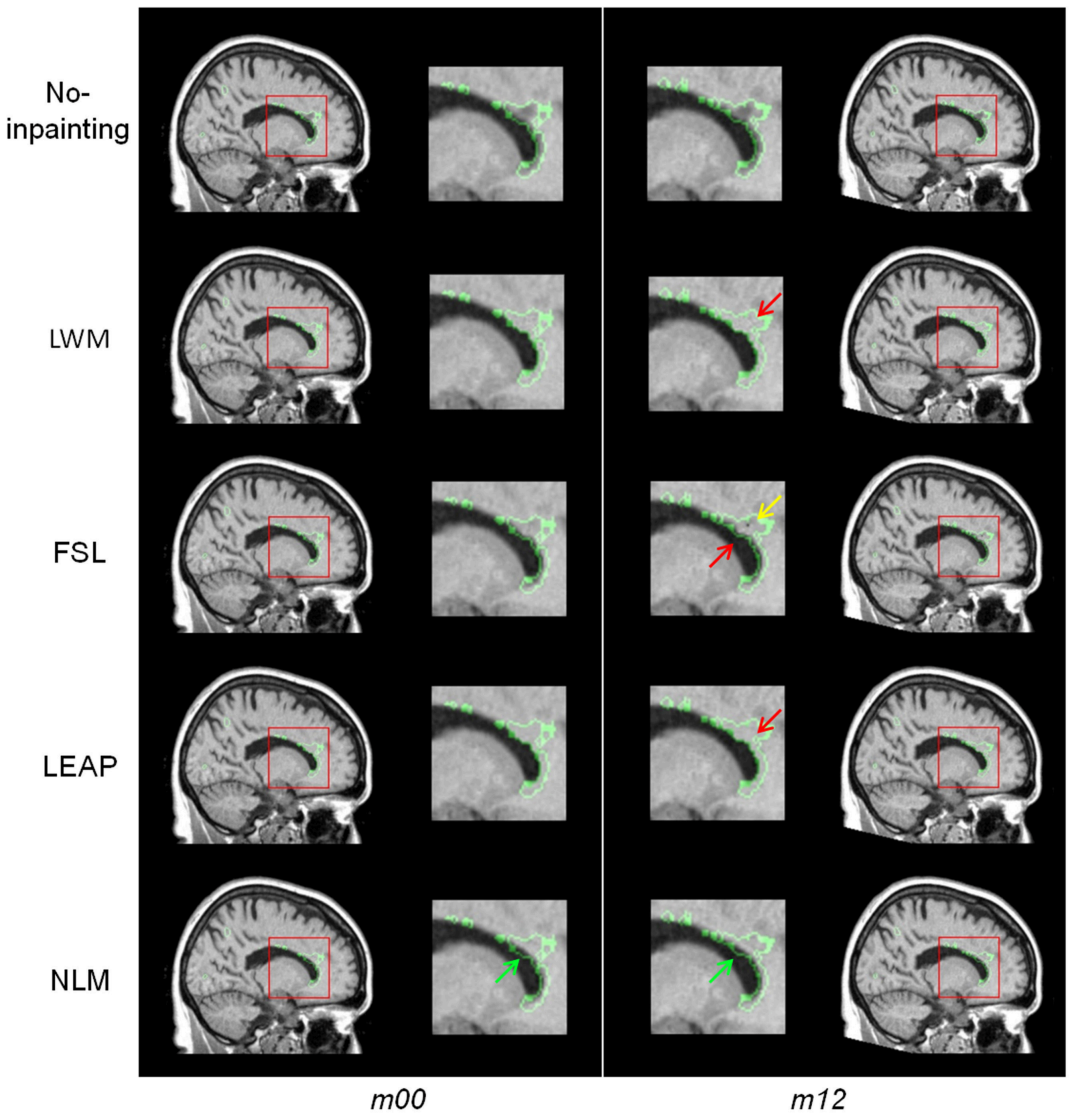
**Example of lesion inpainting on real longitudinal MS data from one patient at baseline (***m00***) and 1 year later (***m12***; first row) for the different methods (LWM, FSL, LEAP, and NLM)**. The red arrows point to anatomically improbable lesion filling results while the green arrow points to plausible and realistic anatomy.

In Figure 7, we show examples of lesion filling on longitudinal data from an MS patient using LWM, FSL, LEAP, and NLM. As can be seen on the original T1W images, the lesion boundary has changed between the two time-points. This is likely affecting the inpainting results, since as discussed previously and described in Figure 5, the performances of LWM, FSL and LEAP are more affected by lesion boundaries than the NLM method. This limitation can be appreciated by comparing the right and left panels of Figure 7, where the extent of the inpainting “bleeding” into ventricles is different for the different time-points. Clearly, this would lead to erroneous longitudinal measures of ventricular enlargement, for example. In contrast, NLM lesion filling seems to provide more plausible contrast and tissue boundaries gradient that are consistent between both time-points (panel *m00* and *m12*).

#### Power analysis of brain atrophy measures

The longitudinal analysis of brain atrophy (PBVC) and ventricular (PVVC) enlargement measurements for all 67 MS subjects using SIENA are summarized in Table 2. The inpainting (LWM, FSL, LEAP, NLM) and the masking strategies resulted in similar mean PBVC and PVVC changes of about −1.1 and 3.8%, respectively. However, NLM has the smallest variability (PBVC *SD* = 0.83% and PVVC *SD* = 4.28%) thus leading to the smallest required sample sizes to detect changes across all assumed treatment effects (10, 30 and 50%). In fact, NLM inpainting leads to a reduction in the number of subjects by a factor of 14% to detect brain volume changes and 21% to detect ventricular enlargement, compared to the volume change estimation on the original data.

**Table 2:** **PBVC and PVVC SIENA results and 10,000 bootstrapping sample size estimation, with a power of 80% and a confidence interval of 95% for different treatment effects (10, 30, and 50%) between ***m00*** and ***m12*****.

**Method**	**PBVC**				**PVVC**			
	**Mean % (*SD*)**	**Sample size**			**Mean % (*SD*)**	**Sample size**		
		**10%**	**30%**	**50%**		**10%**	**30%**	**50%**
Original	–1.12 (0.94)	1171	130	47	3.85 (5.24)	583	65	24
		829–1740	93–196	33–71		329–1145	37–125	13–46
Masked-out	–1.15 (0.98)	1106	123	44	–	–	–	–
		788–1638	98–183	31–66				
LWM	–1.08 (0.95)	1117	124	45	3.76 (4.81)	555	62	22
		806–1661	90–185	32–66		333–1070	37–114	13–42
FSL	–1.13 (0.94)	1153	129	46	3.82 (4.79)	539	60	22
		829–1730	92–193	33–69		316–1026	35–114	13–42
LEAP	–1.12 (0.94)	1179	130	47	3.97 (4.88)	506	56	20
		847–1769	92–193	34–71		308–956	34–108	12–38
NLM	–1.14 (0.83)	**999**	**110**	**40**	3.93 (4.28)	**446**	**49**	**18**
		**763–1389**	**84–152**	**31–56**		**297–727**	**33–2**	**12–29**

*The smallest sample sizes are in bold font. Note, that PVVC cannot be estimated with the Masked-out approach*.

**Figure 8:**
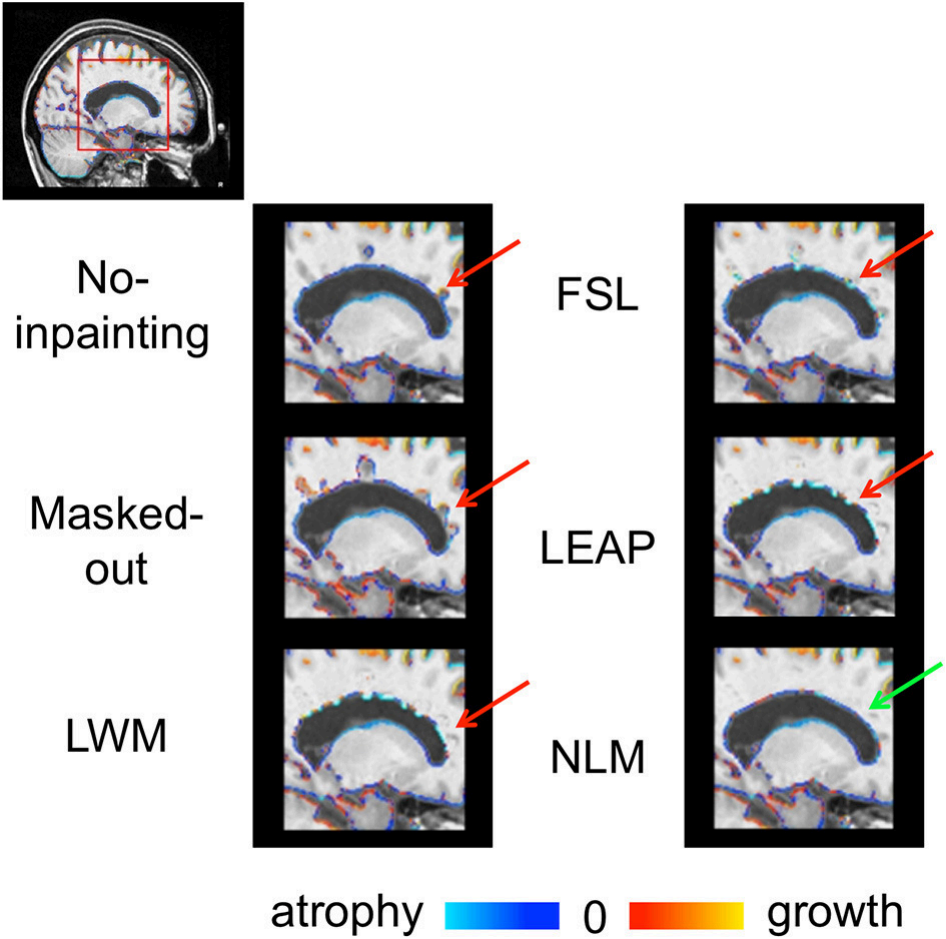
**SIENA brain boundary changes (atrophy = blue and growth red) of the “original” images and with the different strategies to account for lesions (Masked-out, LWM, FSL, LEAP, and NLM)**. The red arrows point to anatomically improbable boundary changes while the green arrow points to plausible and realistic boundary changes.

An example of the SIENA brain boundary change results for one subject can be seen in Figure 8. The figure shows unexpected focal boundary fluctuations (red arrows) in locations where lesions were present on the “original” image without lesion inpainting and with lesion masking but also with WML, FSL, and LEAP lesion inpainting. These changes are particularly visible in regions of larger lesions (e.g., peri-ventricular region). The inpainting approaches reduce these fluctuations but the NLM inpainting results show the most homogenous changes across the boundaries. This likely contributes to the lower variability that this method provides across the whole dataset.

## Discussion

In this work, we propose a new inpainting NLM method to replace MS lesion ROIs with intensities from surrounding normal-appearing brain tissue. We demonstrated the efficiency of our approach in the context of longitudinal image analysis. The proposed approach presents the advantages of not requiring any pre-processing (after lesion identification) and could be applied to any MR image contrast. With MS lesion simulations and with RRMS 1-year longitudinal brain change measures, the results of this study show that the proposed method was superior to the most commonly used inpainting approaches. Furthermore, the qualitative visual results of the proposed approach are realistic and anatomically plausible.

On simulated MS lesions our inpainting approach allows us to reproduce with the best fidelity the original “lesion free” MRI images. Using the NLM operators allows replacing a lesion voxel with voxels having the most similar patches without any explicit *a priori* tissue classification of the healthy tissues. The different boundaries of the lesion masks confirmed this, where bigger masks of the actual lesion do not affect the fidelity of the reconstruction. Therefore, the definition of the lesion mask does not require an accurate definition covering only the affected white matter tissues but a rather “bigger” mask definition. Indeed, because of the propagation strategy using concentric layers, we suggest applying a morphological operator to dilate the lesion mask ROI in order to effectively avoid the propagation of affected tissue intensities.

The evaluation of the different methods in the context of longitudinal brain atrophy and ventricular enlargement measures qualitatively and quantitatively favor the proposed NLM inpainting algorithm. These results suggest that MS lesion inpainting in the context of clinical longitudinal MRI studies have substantial advantages to detect brain atrophy and have already proven to improve some longitudinal structural measurements (Nakamura and Fisher, 2009; Magon et al., 2014). MS lesions are more frequently located in the peri-ventricular region of the brain (Narayanan et al., 1997). This spatial preference could explain the stronger improvement in power to detect ventricular enlargement for the NLM inpainting.

In this study, we only consider WM lesions and T1W images as do most of the inpainting approaches available in MS imaging (LWM, FSL, and LEAP). These approaches require modification of their algorithm to fill GM lesions. But, in MRI studies, GM or cortical lesions have been found in the majority of the RRMS populations (64%; Calabrese et al., 2009). Our method, which does not depend on specific image contrasts, is more flexible in that it can deal with any region where the intensities need to be replaced by intensities from nearby *normal* regions.

We initially proposed to use the NLM as an inpainting operator (Guizard et al., 2013). In this paper, we provide a more thorough validation as well as some improvements on our original approach. Our initial approach inspired Prados et al. (2014) to develop a similar inpainting method based on the NLM, however, their method is different as they only estimate the minimum intensity distance patch before applying a smoothing kernel. Here, we improve on our initial method by proposing a pyramidal hierarchical filling strategy, which enables to capture more structural information at a lower level, propagating this inpainting to the next level. While Prados et al. (2014) search for the most similar patch throughout the whole brain, we show here that inpainting results plateau after at a certain search area distance radius. We believe that the NABT intensities might not be similar across the whole brain, thus limiting the search to a certain distance from the lesion seems adequate. Furthermore, this limited search area reduces the computational burden in comparison to searching over the whole brain area.

On the clinical experiments, the estimated sample size required with SIENA in the current study was smaller (130 with a 30% treatment effect on the original version of SIENA) than previously reported by Anderson et al. (2007) (191 with a 30% treatment effect). These differences could be explained by different factors such as the RRMS population treatment, difference of power (90% for the later) and the study design.

Future work will focus on combining automatic lesion segmentation (Guizard et al., 2015) with the proposed inpainting approach to provide a fully automatic approach. We plan to assess the impact of lesion inpainting in the context of longitudinal non-linear registration and diffusion weighted imaging in order to assess the focal atrophy in the surrounding of the lesion without the confounds due to the presence of lesions.

## Conclusion

We developed a technique to replace tissues of interest, such as MS lesion, with healthy appearing tissues in order to perform cross-sectional and longitudinal image analyses. The method is robust and can improve the statistical power of detecting brain atrophy in MS. Furthermore, the proposed approach does not require any other image pre-processing than the lesion masking.

## Author contributions

All authors contributed equally to this manuscript. NG, KN, PC, VF and DC designed the experiments. NG implemented and analyzed the method and results. DA provided the data.

### Conflict of interest statement

The authors declare that the research was conducted in the absence of any commercial or financial relationships that could be construed as a potential conflict of interest.

## Footnotes

^1^http://fsl.fmrib.ox.ac.uk/fsl/fslwiki/

## References

[B1] Anderson V. M. Bartlett J. W. Fox N. C. Fisniku L. Miller D. H. (2007). Detecting treatment effects on brain atrophy in relapsing remitting multiple sclerosis: sample size estimates. J. Neurol. 254, 1588–1594. 10.1007/s00415-007-0599-3 17940723

[B2] Aubert-Broche B. Griffin M. Pike G. B. Evans A. C. Collins D. L. (2006). Twenty new digital brain phantoms for creation of validation image data bases. IEEE Trans. Med. Imaging 25, 1410–1416. 10.1109/TMI.2006.883453 17117770

[B3] Avants B. B. Epstein C. L. Grossman M. Gee J. C. (2008). Symmetric diffeomorphic image registration with cross-correlation: evaluating automated labeling of elderly and neurodegenerative brain. Med. Image Anal. 12, 26–41. 10.1016/j.media.2007.06.004 17659998 PMC2276735

[B4] Battaglini M. Giorgio A. Stromillo M. Bartolozzi M. Guidi L. Federico A.. (2009). Voxel-wise assessment of progression of regional brain atrophy in relapsing-remitting multiple sclerosis. J. Neurol. Sci. 282, 55–60. 10.1016/j.jns.2009.02.322 19286193

[B5] Battaglini M. Jenkinson M. De Stefano N. (2012). Evaluating and reducing the impact of white matter lesions on brain volume measurements. Hum. Brain Mapp. 33, 2062–2071. 10.1002/hbm.21344 21882300 PMC6870255

[B6] Bertalmío M. Caselles V. Masnou S. Sapiro G. (2014). Inpainting, in Computer Vision, ed Ikeuchi K. (Springer US), 401–416.

[B7] Brett M. (2001). Spatial normalization of brain images with focal lesions using cost function masking. NeuroImage 14, 486–500. 10.1006/nimg.2001.0845 11467921

[B8] Buades A. Coll B. Morel J. M. (2005). A non-local algorithm for image denoising. Comput. Vision Pattern Recogn. 2, 60–65. 10.1109/cvpr.2005.38

[B9] Calabrese M. F. Agosta F. Rinaldi, I. Mattisi, P. Grossi, A. Favaretto, M.. (2009). Cortical lesions and atrophy associated with cognitive impairment in relapsing-remitting multiple sclerosis. Arch. Neurol. 66, 1144–1150. 10.1001/archneurol.2009.174 19752305

[B10] Ceccarelli A. Jackson J. S. Tauhid S. Arora A. Gorky J. Dell'Oglio E.. (2012). The impact of lesion in-painting and registration methods on voxel-based morphometry in detecting regional cerebral gray matter atrophy in multiple sclerosis. AJNR Am. J. Neuroradiol. 33, 1579–1585. 10.3174/ajnr.A3083 22460341 PMC3425668

[B11] Chard D. T. Jackson J. S. Miller D. H. Wheeler-Kingshott C. A. M. (2010). Reducing the impact of white matter lesions on automated measures of brain gray and white matter volumes. J. Magn. Reson. Imaging 32, 223–228. 10.1002/jmri.22214 20575080

[B12] Collins D. L. Neelin P. Peters T. M. Evans A. C. (1994). Automatic 3D intersubject registration of MR volumetric data in standardized Talairach space. J. Comput. Assist. Tomogr. 18, 192–205. 10.1097/00004728-199403000-00005 8126267

[B13] Collins D. L. Zijdenbos A. P. Kollokian V. Sled J. G. Kabani N. J. Holmes C. J.. (1998). Design and construction of a realistic digital brain phantom. IEEE Trans. Med. Imaging 17, 463–468. 10.1109/42.712135 9735909

[B14] Criminisi A. Perez P. Toyama K. (2004). Region filling and object removal by exemplar-based image inpainting. IEEE Trans. Image Process. 13, 1200–1212. 10.1109/TIP.2004.833105 15449582

[B15] Derakhshan M. Caramanos Z. Giacomini P. S. Narayanan S. Maranzano J. Francis S. J.. (2010). Evaluation of automated techniques for the quantification of grey matter atrophy in patients with multiple sclerosis. Neuroimage 52, 1261–1267. 10.1016/j.neuroimage.2010.05.029 20483380

[B16] Efros A. A. Leung T. K. (1999). Texture synthesis by non-parametric sampling, in International Conference on Computer Vision: ICCV 2, Vol. 1032 (Kerkyra). 10.1109/iccv.1999.790383

[B17] Fadili M.-J. Starck J.-L. Murtagh F. (2009). Inpainting and zooming using sparse representations. Comp. J. 52, 64–79. 10.1093/comjnl/bxm055

[B18] Fazekas F. Barkhof F. Filippi M. Grossman R. I. Li D. K. McDonald W. I.. (1999). The contribution of magnetic resonance imaging to the diagnosis of multiple sclerosis. Neurology 53, 448–456. 10.1212/WNL.53.3.448 10449103

[B19] Francis S. J. (2004). Automatic Lesion Identification in MRI of Multiple Sclerosis Patients. Master Thesis, McGill University, Montreal, QC.

[B20] Guizard N. Coupé P. Fonov V. S. Manjón J. V. Arnold D. L. Collins D. L. (2015). Rotation-invariant multi-contrast non-local means for MS lesion segmentation. NeuroImage 8, 376–389. 10.1016/j.nicl.2015.05.001 26106563 PMC4474283

[B21] Guizard N. Nakamura K. Coupé P. Arnold D. L. Collins D. L. (2013). Non-local MS MRI lesion inpainting method for image processing, in The EndMS Conference (Saint-Sauveur, QC).

[B22] Lansley J. Mataix-Cols D. Grau M. Radua J. Sastre-Garriga J. (2013). Localized grey matter atrophy in multiple sclerosis: a meta-analysis of voxel-based morphometry studies and associations with functional disability. Neurosci. Biobehav. Rev. 37, 819–830. 10.1016/j.neubiorev.2013.03.006 23518268

[B23] Liu Y. Caselles V. (2013). Exemplar-based image inpainting using multiscale graph cuts. IEEE Trans. Image Process. 22, 1699–1711. 10.1109/TIP.2012.2218828 22997270

[B24] Magon S. Gaetano L. Chakravarty M. M. Lerch J. Naegelin Y. Stippich C.. (2014). White matter lesion filling improves the accuracy of cortical thickness measurements in multiple sclerosis patients: a longitudinal study. BMC Neurosci. 15:106. 10.1186/1471-2202-15-106 25200127 PMC4164794

[B25] Meier D. Fisher E. (2005). Atlas-based anatomic labeling in neurodegenerative disease via structure-driven atlas warping. J. Neuroimaging 15, 16–26. 10.1111/j.1552-6569.2005.tb00281.x 15574570

[B26] Nakamura K. Fisher E. (2009). Segmentation of brain magnetic resonance images for measurement of gray matter atrophy in multiple sclerosis patients. Neuroimage 44, 769–776. 10.1016/j.neuroimage.2008.09.059 19007895 PMC3001325

[B27] Nakamura K. Guizard N. Fonov V. Narayanan S. Collins L. Arnold D. (2014). Jacobian integration method increases the statistical power to measure gray matter atrophy in multiple sclerosis. Neuroimage Clin. 4, 10–17. 10.1016/j.nicl.2013.10.015 24266007 PMC3830061

[B28] Narayanan S. Fu L. Pioro E. De Stefano N. Collins D. L. Francis G. S.. (1997). Imaging of axonal damage in multiple sclerosis: spatial distribution of magnetic resonance imaging lesions. Ann. Neurol. 41, 385–391. 10.1002/ana.410410314 9066360

[B29] Popescu V. Ran N. C. Barkhof F. Chard D. T. Wheeler-Kingshott C. A. Vrenken H. (2014). Accurate GM atrophy quantification in MS using lesion-filling with co-registered 2D lesion masks. Neuroimage Clin. 4, 366–373. 10.1016/j.nicl.2014.01.004 24567908 PMC3930097

[B30] Prados F. Cardoso M. J. MacManus D. Wheeler-Kingshott C. Ourselin S. (2014). A modality-agnostic patch-based technique for lesion filling in multiple sclerosis. Med. Image Comput. Comp. Assist. Interv. 17(Pt 2), 781–788. 10.1007/978-3-319-10470-6_97 25485451

[B31] Prinster A. Quarantelli M. Orefice G. Lanzillo R. Brunetti A. Mollica C.. (2006). Grey matter loss in relapsing–remitting multiple sclerosis: a voxel-based morphometry study. Neuroimage 29, 859–867. 10.1016/j.neuroimage.2005.08.034 16203159

[B32] Rovira A. Auger C. Alonso J. (2013). Magnetic resonance monitoring of lesion evolution in multiple sclerosis. Ther. Adv. Neurol. Disord. 6, 298–310. 10.1177/1756285613484079 23997815 PMC3755529

[B33] Rudick R. A. Fisher E. Lee J. C. Simon J. Jacobs L. G. (1999). The multiple sclerosis collaborative research use of the brain parenchymal fraction to measure whole brain atrophy in relapsing-remitting MS. Neurology 53, 1698–1698. 10.1212/WNL.53.8.1698 10563615

[B34] Sanfilipo M. Benedict R. Weinstock-Guttman B. Bakshi R. (2006). Gray and white matter brain atrophy and neuropsychological impairment in multiple sclerosis. Neurology 66, 685–692. 10.1212/01.wnl.0000201238.93586.d9 16534104

[B35] Sdika M. Pelletier D. (2009). Nonrigid registration of multiple sclerosis brain images using lesion inpainting for morphometry or lesion mapping. Hum. Brain Mapp. 30, 1060–1067. 10.1002/hbm.20566 18412131 PMC6870756

[B36] Sled J. G. Zijdenbos A. P. Evans A. C. (1998). A nonparametric method for automatic correction of intensity nonuniformity in MRI data. IEEE Trans. Med. Imaging 17, 87–97. 10.1109/42.668698 9617910

[B37] Smith S. (2002). Fast robust automated brain extraction. Hum. Brain Mapp. 17, 143–155. 10.1002/hbm.10062 12391568 PMC6871816

[B38] Smith S. Zhang Y. Jenkinson M. Chen J. Matthews P. M. Federico A.. (2002). Accurate, robust, and automated longitudinal and cross-sectional brain change analysis. Neuroimage 17, 479–489. 10.1006/nimg.2002.1040 12482100

[B39] Tao G. Datta S. He R. Nelson F. Wolinsky J. Narayana P. (2009). Deep gray matter atrophy in multiple sclerosis: a tensor based morphometry. J. Neurol. Sci. 282, 39–46. 10.1016/j.jns.2008.12.035 19168189 PMC2744867

[B40] Telea A. (2004). An image inpainting technique based on the fast marching method. J. Graphics Tools 9, 23–34. 10.1080/10867651.2004.10487596

[B41] Vrenken H. Geurts J. J. Knol D. L. Polman C. H. Castelijns J. A. Pouwels P. J.. (2006). Normal-appearing white matter changes vary with distance to lesions in multiple sclerosis. Am. J. Neuroradiol. 27, 2005–2011. 17032884 PMC7977884

[B42] VQEG V. Q. E. (2003). Final Report from the Video Quality Experts Group on the Validation of Objective Models of Video Quality Assessment, Phase II (FR_TV2).

[B43] Wong A. Orchard J. (2008). A nonlocal-means approach to exemplar-based inpainting, in International Conference on Image Processing (San Diego, CA: IEEE). 10.1109/icip.2008.4712326

[B44] Zhang Y. Brady M. Smith S. (2001). Segmentation of brain MR images through a hidden Markov random field model and the expectation-maximization algorithm. IEEE Trans. Med. Imaging 20, 45–57. 10.1109/42.906424 11293691

[B45] Zijdenbos A. Forghani R. Evans A. (1998). Automatic quantification of MS Lesions in 3D MRI brain data sets: validation of INSECT, in Medical Image Computing and Computer-Assisted Interventation—MICCAI'98, eds Wells W. M. Colchester A. Delp S. (Cambridge, MA; Berlin; Heidelberg: Springer), 439–448.

